# Effect of Washing, Waxing and Low-Temperature Storage on the Postharvest Microbiome of Apple

**DOI:** 10.3390/microorganisms8060944

**Published:** 2020-06-23

**Authors:** Ahmed Abdelfattah, Susan R. Whitehead, Dumitru Macarisin, Jia Liu, Erik Burchard, Shiri Freilich, Christopher Dardick, Samir Droby, Michael Wisniewski

**Affiliations:** 1 Institute of Environmental Biotechnology, Graz University of Technology, Petersgasse 12, 8010 Graz, Austria; 2 Department of Ecology, Environment and Plant Sciences, University of Stockholm, Svante Arrhenius väg 20A, 11418 Stockholm, Sweden; 3 Department of Biological Sciences, Virginia Polytechnic Institute and State University, 220 Ag Quad Ln, Blacksburg, VA 24061, USA; 4 U.S. Food and Drug Administration, 501 Campus Drive, College Park, MD 20740, USA; Dumitru.Macarisin@fda.hhs.gov; 5 Chongqing Key Laboratory of Economic Plant Biotechnology, College of Landscape Architecture and Life Sciences, Chongqing University of Arts and Sciences, Yongchuan, Chongqing 402160, China; liu.jia1983@hotmail.com; 6 U.S. Department of Agriculture—Agricultural Research Service, 2217 Wiltshire Road, Kearneysville, WV 25430, USA; Erik.Burchard@ars.usda.gov (E.B.); chris.dardick@usda.gov (C.D.); 7 Institute of Plant Sciences, Newe Ya’ar Research Center, The Agricultural Research Organization, Ramat Yishay 30095, Israel; shiri.freilich@gmail.com; 8 Department of Postharvest Science, Agricultural Research Organization, The Volcani Center, Rishon LeZion 7505101, Israel; samird@volcani.agri.gov.il

**Keywords:** microbial composition, foodborne pathogens, postharvest management, fruit microbiome, microbiota, carposphere, *Malus domestica*, Empire apples, plant microbiota

## Abstract

There is growing recognition of the role that the microbiome plays in the health and physiology of many plant species. However, considerably less research has been conducted on the postharvest microbiome of produce and the impact that postharvest processing may have on its composition. Here, amplicon sequencing was used to study the effect of washing, waxing, and low-temperature storage at 2 °C for six months on the bacterial and fungal communities of apple calyx-end, stem-end, and peel tissues. The results of the present work reveal that tissue-type is the main factor defining fungal and bacterial diversity and community composition on apple fruit. Both postharvest treatments and low temperature storage had a strong impact on the fungal and bacterial diversity and community composition of these tissue types. Distinct spatial and temporal changes in the composition and diversity of the microbiota were observed in response to various postharvest management practices. The greatest impact was attributed to sanitation practices with major differences among unwashed, washed and washed-waxed apples. The magnitude of the differences, however, was tissue-specific, with the greatest impact occurring on peel tissues. Temporally, the largest shift occurred during the first two months of low-temperature storage, although fungi were more affected by storage time than bacteria. In general, fungi and bacteria were impacted equally by sanitation practices, especially the epiphytic microflora of peel tissues. This research provides a foundation for understanding the impact of postharvest management practices on the microbiome of apple and its potential subsequent effects on postharvest disease management and food safety.

## 1. Introduction

There is growing recognition that microbiota play a critical role in the health and physiology of their hosts [1,2]. In fact, many organisms may be inseparable from their microbiota and are best viewed as metaorganisms [3]. In humans, microbes can impact many aspects of health, and their composition is highly variable among individuals [4]. Regarding plants, studies of the rhizosphere microbiome have clearly demonstrated the beneficial role that the microbiome has on growth, development, as well as abiotic and biotic stress resistance [5,6,7,8]. While numerous studies have been conducted on the rhizosphere microbiome and its impact on the physiology of its host, less research has been conducted on the microbiome of harvested crops [9,10,11,12]. This is despite the fact that the microbiome of harvested produce will be ingested by humans, where it can potentially have a significant impact on human health. The microbiota of harvested produce is especially relevant to issues of food safety and the harboring of foodborne pathogens [10,13].

The storage and shelf-life of harvested produce can be defined as the length of time harvested produce can be stored and subsequently marketed while retaining good quality indices, such as appearance, taste, texture, safety, and nutritional value. Common practices used to increase apple storage and shelf life include precooling, washing and sanitizing, and waxing. These simple treatments allow apples to be stored for 6–12 months at low temperatures (1–2 °C) until they are shipped to distributors and then to supermarkets. While fruit washing is intended to remove unwanted debris and reduce microbial load, it can also impact the natural protective wax layer that covers a fruit surface. For this reason, it is a common practice to apply a thin layer of edible wax, typically made of esters of a higher fatty acid with monohydric alcohols, hydrocarbons, and some free fatty acids, to serve the same function as natural wax, i.e., covering fruit injuries, reducing water loss and/or shrinking following fruit washing, and adding a shine/gloss to the fruit surface.

Previous research revealed spatial and temporal changes in the composition of the microbiome of different portions (peel, wound, calyx-end, stem-end) of apple fruit obtained at the point of consumer purchase (supermarket) and representing fruit grown using different approaches (organic vs. conventional) [10]. Similar research was recently conducted that focused on the bacterial composition of harvested apples in different fruit tissues and the impact of organic vs. conventional management practices [14]. These studies, however, could not discern when particular components of the microbiome became established or were altered, or the impact that different postharvest processing procedures had on the composition of the microbiome. For instance, a high prevalence of yeast genera (*Malassezia*, *Candida*, and *Trichosporon*) associated with dermal disorders in humans was observed on apples purchased at a supermarket, but the source of these genera from handling prior to or after arriving at the supermarket could not be determined [10]. The composition of the postharvest microbiome of apple can also be impacted by bacterial and fungal communities present in packing houses that process fruit for market and storage after harvest. A recent study reported substantial differences in both the composition and diversity of the environmental microbiota present in different tree fruit processing facilities, and revealed that the presence of *Pseudomonadaceae* and *Diplodascaceae* families was associated with an increased occurrence and persistence of the foodborne pathogen *Listeria monocytogenes* [15]. Further research on this topic may lead to a better understanding of the interactions that occur between pathogenic and nonpathogenic microorganisms found on harvested fruits and the optimization of fruit handling strategies. A strong effect of apple genotype on the composition of the endophytic microbiome of apple stem tissues and rootstocks has also been reported [16].

The current study was designed to (1) evaluate the impact of postharvest washing and waxing on the microbial community associated with apple peel, stem-end, and calyx-end tissues, and (2) determine the impact of storage time over a period of six months at low temperature (2 °C) on the microbial community of the same apple tissues. Amplicon sequencing of the bacterial 16S and fungal ITS region was used to analyze and compare the microbial communities of unwashed (UW), washed (W), and washed-waxed (WW) apple peel, calyx-end, and stem-end tissues.

The present study is part of larger project whose objectives are to define a core apple microbiome, assess the effects of management practices, including the use of postharvest biocontrol agents on the composition of the apple microbiome, and investigate potential interactions occurring between members of the microbiome and the interaction between fruit tissues and the resident microbiome [17,18,19]. The overall objective is to develop a science-based strategy for manipulating the apple microbiome to prevent postharvest decay and physiological disorders.

## 2. Materials and Methods

### 2.1. Harvesting of Apples, Treatments and Experimental Design

Apple fruit (cv. ‘Enterprise’) were harvested from an orchard block located at the U.S. Department of Agriculture—Agricultural Research Service (USDA-ARS) in Kearneysville, WV. Apples were harvested in October, 2016, and then divided into three treatments: unwashed (UW), washed (W), and washed and waxed (WW). While UW fruit were placed directly into storage in tray-packed boxes, W and WW were submersed in 0.015% hypochlorite solution, rinsed in water, and dried before separating them into two groups. Fruits from the first group of the washed apples were coated with Shield-Brite^®^ AP-40 wax (Pace International, Wapato, WA, USA), a wax that is commonly used in the local region. The wax was applied at room temperature as a fine mist using a spray bottle, as previously described [20]. Specifically, each apple was sprayed with five trigger pulls, i.e., one pull each was applied to the stem and calyx ends, and three pulls to coat the rest of the apple, then dried at 23 °C for 30 min. Fruits from the three treatments placed into tray-pack cartons and stored at 2 °C, and then sampled at 0 (before storage), 2, 4, and 6 months of storage. All apples were harvested and all treatments were applied to the harvested apples which were then placed into storage on the same day.

**Figure 1:**
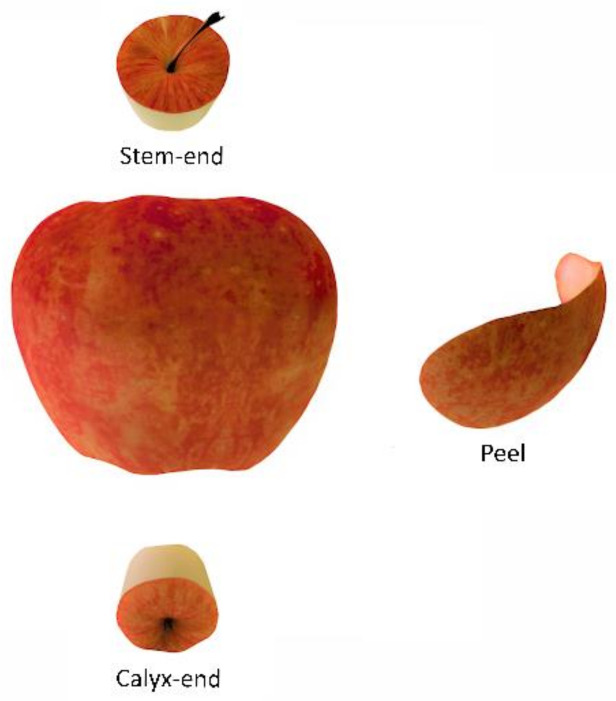
Schematic representation of the different apple tissues i.e., stem-end, calyx-end, and peel sampled and analyzed in the present study.

Sampling consisted of collecting three tissue types pooled from 3 apples per biological replicate (Figure 1). Peel tissue was collected using a vegetable peeler to remove peel from the hemisphere of each fruit. Calyx and stem tissue were collected by coring the apple from top to bottom and removing and sectioning the calyx and stem ends with a razor blade, as previously described [10]. All necessary tools were washed and surface sterilized with 70% ethanol between sampling, and all samples were immediately frozen in liquid nitrogen prior to being stored at −80 °C. In summary, the total number of samples (*n* = 288 samples) consisted of 3 treatments × 4 sampling points × 3 tissues × 8 replicates, and each replicate sample was a composite of the same tissue type pooled from 3 fruits.

### 2.2. Library Preparation and Sequencing

All samples were ground in liquid nitrogen and homogenized prior to DNA extraction using a DNeasy PowerLyzer PowerSoil Kit (Qiagen, Germantown, MD, USA). Initial tissue disruption of 250 mg was performed with a Qiagen PowerLyzer 24 Homogenizer (Qiagen, Germantown, MD, USA). DNA extractions were automated using a Qiagen QiaCube (Qiagen, Germantown, MS, USA), using the processing routine recommended by the manufacturer of the PowerSoil kit (Qiagen, Germantown, MS, USA). Extracted DNA was used as the template for amplicon PCR reactions that amplified the bacterial 16S ribosomal region and the fungal internal transcribed spacer (ITS) region; 16S amplicons were amplified using the universal primers 515F [21] and 806R [22] in conjunction with peptide nucleic acids (PNAs) (PNA Bio, CA, USA) added to inhibit amplification of ribosomal and mitochondrial sequences [23]. ITS amplicons were amplified using ITS3/KYO2 [24] and ITS4 [25] primers, along with a custom-designed blocking oligo designed to inhibit amplification of the host DNA (5′ ATTGATATGCTTAAATTCAGCGGGTAACCCCGCCTGACCTGGGGTCGCGTT-C3 spacer 3′). All primers were modified to include the necessary Illumina adapters (www.illumina.com) for subsequent PCR addition of Illumina indexes for multiplexing.

For bacterial (16S) amplicon generation, PCR reactions were conducted in a total volume of 25 μL, containing 12.5 μL of KAPA HiFi HotStart ReadyMix (Sigma-Aldrich, St. Louis, MO, USA), 1.0 μL of each primer (10 μM), 2.5 μL of mitochondrial PNA (5 μM), 2.5 μL of plastid PNA (5 μM), 2.5 μL of DNA template, and 3 μL nuclease-free water. Reactions were incubated in a T100 thermal cycler (BioRad, Hercules, CA, USA) at 95 °C for 5 min followed by 30 cycles of 95 °C for 30 s, 78 °C for 5 s, 55 °C for 30 s, and 72 °C for 30 s, concluding with a final extension at 72 °C for 5 min. For fungal (ITS) amplicon generation, 25 μL PCR reactions contained 12.5 μL of KAPA HiFi HotStart ReadyMix (Kapa Biosystems), 1.0 μL of each primer (10 μM), 1.0 μL of blocking oligo (10 μM), 2.5 μL of DNA template, and 7 μL nuclease-free water. Reactions were incubated in a T100 thermal cycler (BioRad) at 95 °C for 5 min followed by 30 cycles of 95 °C for 30 s, 55 °C for 30 s, and 72 °C for 30 s, concluding with a final extension at 72 °C for 5 min. Nuclease-free water (Qiagen, Valencia, CA, USA) replaced template DNA in negative controls. Library preparation following amplicon PCR was performed as specified in the Illumina 16s Metagenomic Sequencing Library Preparation guide, in conjunction with the use of a Nextera Index Kit (Illumina, San Diego, CA, USA) containing 96 indexes. Subsequent library size, quality, and confirmation of the absence of adapter dimers was performed on an Agilent 2100 Bioanalyzer (Agilent, Santa Clara, CA, USA). Paired-end sequencing of amplicons, including negative controls, was done on an Illumina MiSeq (Illumina) sequencer with a V3 600-cycle Reagent Kit (Illumina).

### 2.3. Bioinformatic and Statistical Analyses

Sequence demultiplexing, quality and chimeric trimming, generation of Amplicon Sequence Variant (ASV) table, and rarefaction to account for uneven sequencing depth was done using the default parameters in DADA2 [26] as implemented in Qiime2 [27]. Taxonomic clustering of ASVs was done using a similarity threshold of 97% against the GreenGenes [28] database for 16S reads and against the UNITE [29] database for ITS reads. MetagenomeSeq’s Cumulative Sum Scaling (CSS) [30] was used as a normalization method for subsequent community composition analyses, including the calculation of Bray–Curtis dissimilarity metrics [31], the construction of PCoA plots, and PERMANOVA analyses.

To assess the effects of postharvest treatment and storage on the diversity of the apple microbiome across different tissue types, we used a series of ANOVA and adonis (~PERMANOVA) models with Shannon diversity and community composition as the response variables, respectively. Separate analyses were conducted for bacteria and fungi. In initial analyses, postharvest treatment (UW, W or WW), storage time (0, 2, 4, or 6 months postharvest, modeled as a continuous variable in the case of Shannon diversity), tissue type (stem, calyx, or peel), and all two- and three-way interactions among these variables were used as predictor variables. Based on results showing numerous two- and three-way interactions involving tissue type, separate analyses for each tissue type were conducted. Here, treatment, storage time, and their interaction were included as predictors. In cases where significant interactions were found, the data were further separated by postharvest treatment regime to examine the effects of storage time on the microbial diversity for each treatment. Similarly, in cases where we detected significant effects of categorical predictor variables (e.g., treatment), we conducted Tukey’s post hoc tests in the case of Shannon diversity, and pairwise adonis (~PERMANOVA) [32] in the case of community composition analysis, to assess specific differences among groups. All analyses were conducted using R version 3.6.2 in RStudio version 1.1.453 and the packages vegan version 2.5-6, lme4 version 1.1-21, multcomp version 1.4-13, phyloseq version 1.32.0 [32,33,34,35,36].

Taxonomic heat trees were generated using the Metacoder [37] package in R to identify and visualize overall abundance, as well as differential abundance among postharvest treatments and sampling points. The differential abundance analysis was based on pairwise comparisons of the log-2 ratio of median proportions of the reads observed in each treatment and at each sampling point. Only significant differences are colored in the resulting trees, determined using a Wilcox rank-sum test followed by a Benjamini-Hochberg (FDR) correction for multiple comparisons. In addition, the most prevalent taxa, which had a relative abundance >0.1% across all samples, were used to evaluate differences in the relative abundance of the detected taxa between tissue type and treatments using the Kruskal–Wallis method [38].

## 3. Results

### 3.1. Sequencing Results

A total of 5,757,276 reads were obtained for the 16S data and assigned to 2357 ASVs after paired-end alignments, quality filtering, and deletion of chimeric, singletons, and nontarget plant sequences. Broken down by tissue-type, the number of 16S sequences ranged from 596–137,861 in for calyx-end, 130–14,236 in peel, and 2361–340 for stem-end tissue (Table S1). A total of 264 of the 288 samples were used in the subsequent analyses after rarefying to an even depth of 669 reads per sample. In contrast, a much greater number of ITS sequences was obtained. In total, 20,057,710 reads were obtained for the ITS data and assigned to 5015 ASVs after paired-end alignments, quality filtering, and deletion of chimeric, singletons, and nontarget plant sequences. A range of 4927–808,548 reads was obtained for calyx tissue, 3189–173,795 for peel tissue, and 3558–732,726 for stem tissues. Similar to the 16S data, the smallest number of reads was obtained from peel tissue; however, no major differences were observed between stem and calyx tissue. A total of 281 of 288 samples were used in the subsequent analyses after rarefying to an even depth of 5015 reads per sample (Table S1).

### 3.2. Bacterial and Fungal Communities Associated with Apple Calyx-End, Stem-End, and Peel Tissues

**Figure 2:**
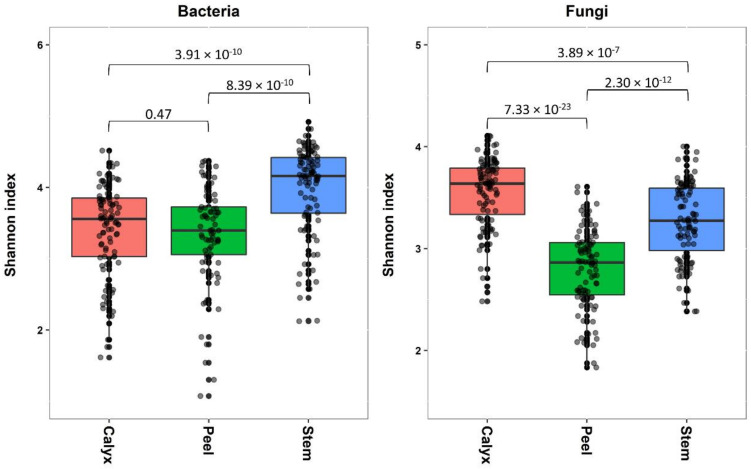
Box plots showing the bacterial fungal diversity (Shannon index) in the apple calyx-end, peel, and stem-end tissues. Superimposed on the box plots are the horizontally jittered raw data points. Values indicate *p* values of the results of pairwise comparison using nonparametric *t*-test.

Tissue type had a significant effect on bacterial and fungal diversity, according to Shannon index (Table 1). While the bacterial diversity present in stem-end was significantly higher compared to those in peel and calyx-end tissues, both peel and calyx end tissues had similar diversity levels (Figure 2). In contrast, fungal diversity differed significantly between all tissue types, with the highest fungal diversity being found in calyx-end, followed by stem-end, and peel tissue (Figure 2).

**Figure 3:**
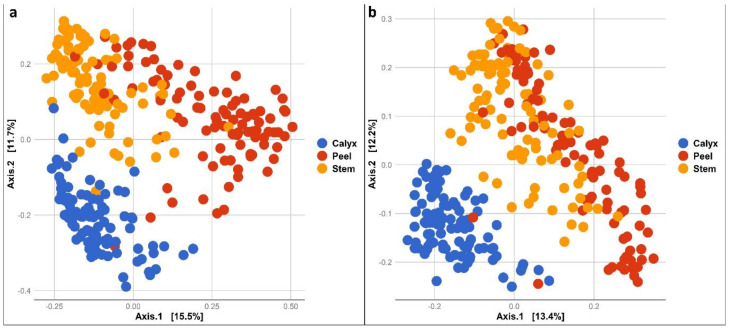
PCoA based on Bray Curtis dissimilarity metrics, showing the distance in the bacterial (**a**) and fungal (**b**) communities between apple calyx-end, peel, and stem-end tissues.

In addition, bacterial and fungal community composition differed significantly among apple peel, stem-end, and calyx-end tissues (PERMANOVA bacteria: *R*^2^ = 0.19293, *p* = 0.001 and fungi: *R*^2^ = 0.16309, *p* = 0.001). This was also evident in pairwise PERMANOVAs comparing composition among different tissue types for both fungal and bacterial communities (Table S2). A Principal Coordinate Analysis (PCoA) of the bacterial community illustrates the distinct bacterial community composition in the different tissue types (Figure 3a). Similarly, the fungal community composition of different tissue types appeared to form separate clusters, although peel and stem-end tissues overlapped (Figure 3b). The differences in community composition among the tissue types were also observed in all treatments at each sampling point during storage (Figure S1).

**Figure 4:**
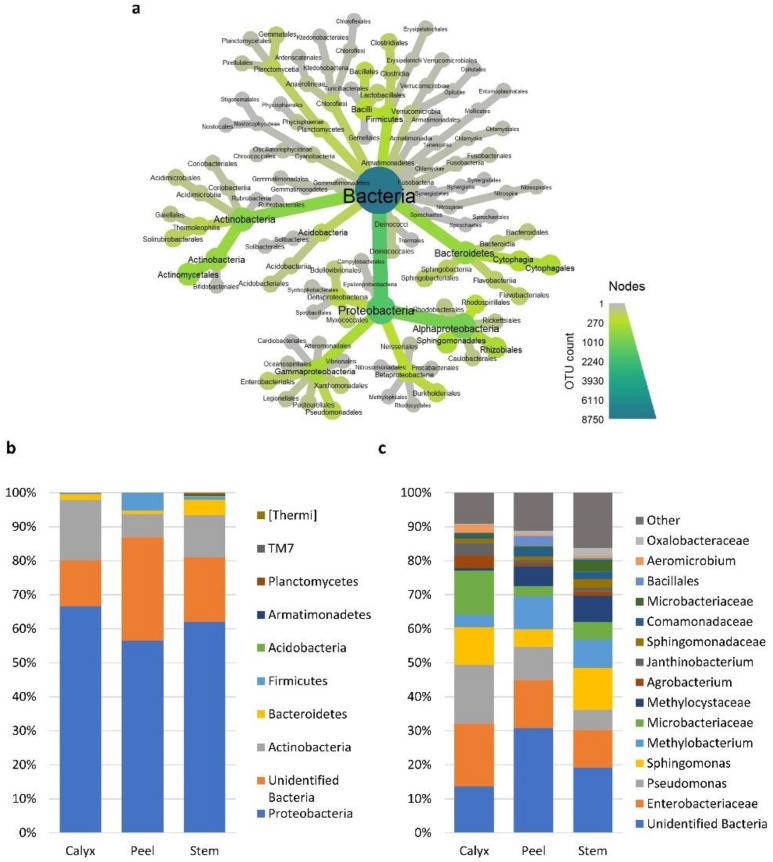
Relative abundances of the most prevalent bacterial taxa >1%. (**a**) a heat tree showing the overall taxonomic hierarchy from phyla to order level. The size and color of nodes and edges are correlated with taxa abundance across all the investigated samples. (**b**) bar plot showing the distribution of the bacterial phyla among apple tissue types (calyx-end, peel, and stem-end tissues), and (**c**) at the lowest identifiable taxonomical level. Taxa with relative abundance less than 1% were merged into “Other” group in grey.

The observed differences in diversity among tissue types were due to changes in the presence/absence of taxa in different tissues and/or significant changes in the relative abundance of specific taxa (Table S3). At the bacterial phylum level, *Firmicutes* and an unidentified bacterial phylum had a much higher relative abundance in peel tissues relative to calyx-end and stem-end tissues. Stem-end tissues had a higher number of bacterial phyla, including *Acidobacteria*, *Armatimonadetes*, *Planctomycetes*, *TM7*, and *Thermi* relative to other tissues (Figure 4a,b).

**Figure 5:**
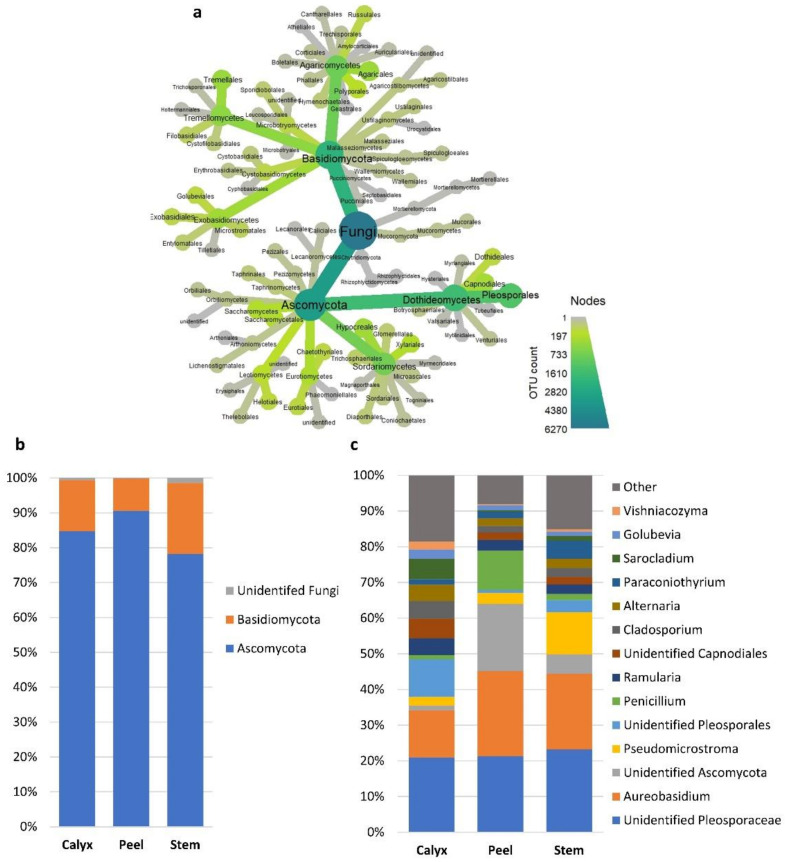
Relative abundances of the most prevalent fungal taxa >1%. (**a**) a heat tree showing the overall taxonomic hierarchy from phyla to order level. The size and color of nodes and edges are correlated with taxa abundance across all the investigated samples. (**b**) bar plot showing the distribution of the fungal phyla among apple tissue types (calyx-end, peel, and stem-end tissues), and (**c**) at the Lowest identifiable taxonomical level. Taxa with relative abundance less than 1% were merged together into “Other” group in grey.

Bacteria families/genera such as *Microbacteriaceae*, *Pseudomonas*, and *Enterobacteriaceae* had a higher abundance in calyx-end tissues. In contrast, peel tissue had a higher level of unidentified bacteria (Figure 4c and Table S3). At the fungal phylum level, all three tissue types had relatively similar distribution between ascomycete and basidiomycete fungi, although differences at the genus level were more evident (Figure 5a–c). For example, *Penicillium* and an identified genus in the phylum *Ascomycota* had a higher abundance in peel tissue than in other tissues (Figure 5c). In calyx-end tissue, unidentified *Pleosporaceae*, and *Sarocladium* were more dominant, while *Pseudomicrostroma* and *Paraconiothyrium* were more prevalent in stem-end tissue (Figure 5c and Table S3).

### 3.3. Effect of Washing and Waxing and Time of Storage on Apple Peel, Stem-End, and Calyx-End Tissues

#### 3.3.1. Shannon Diversity

**Figure 6:**
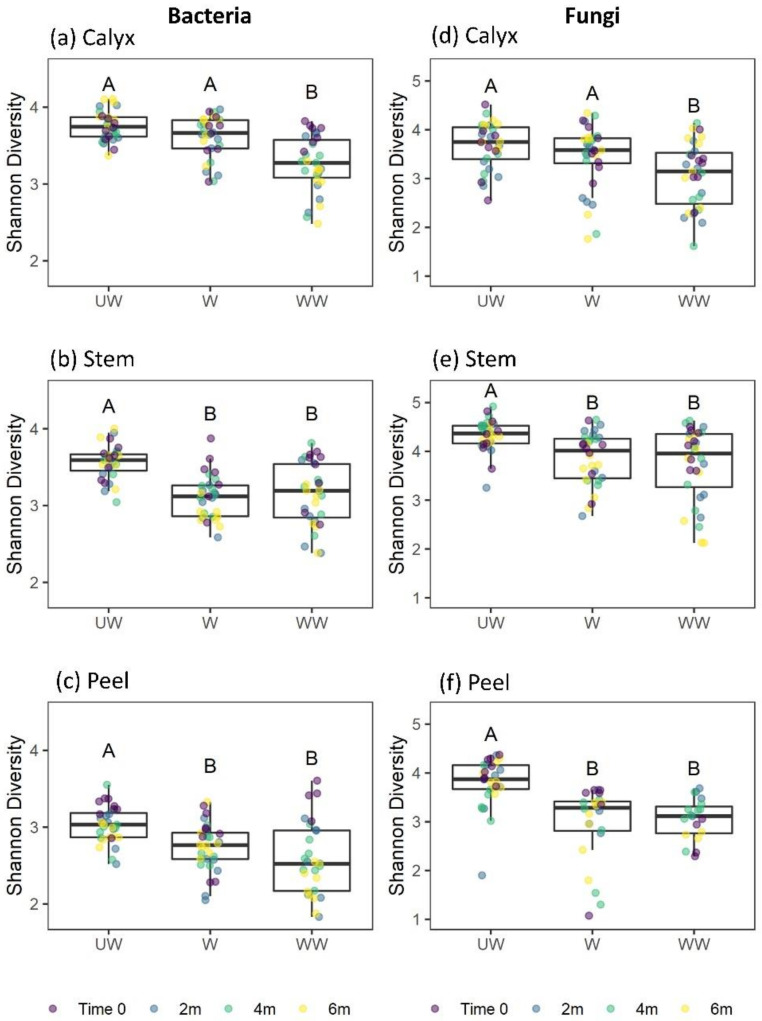
Box plots showing the bacterial and fungal diversity (Shannon index) in the apple calyx-end (**a**,**d**), stem-end (**b**,**e**), and peel (**c**,**f**) tissues. Superimposed on the box plots are the horizontally jittered raw data points colored according to the sampling points (0, 2 m, 4 m, and 6 m). Different letters indicate significant differences between groups (*p* < 0.05).

**Figure 7:**
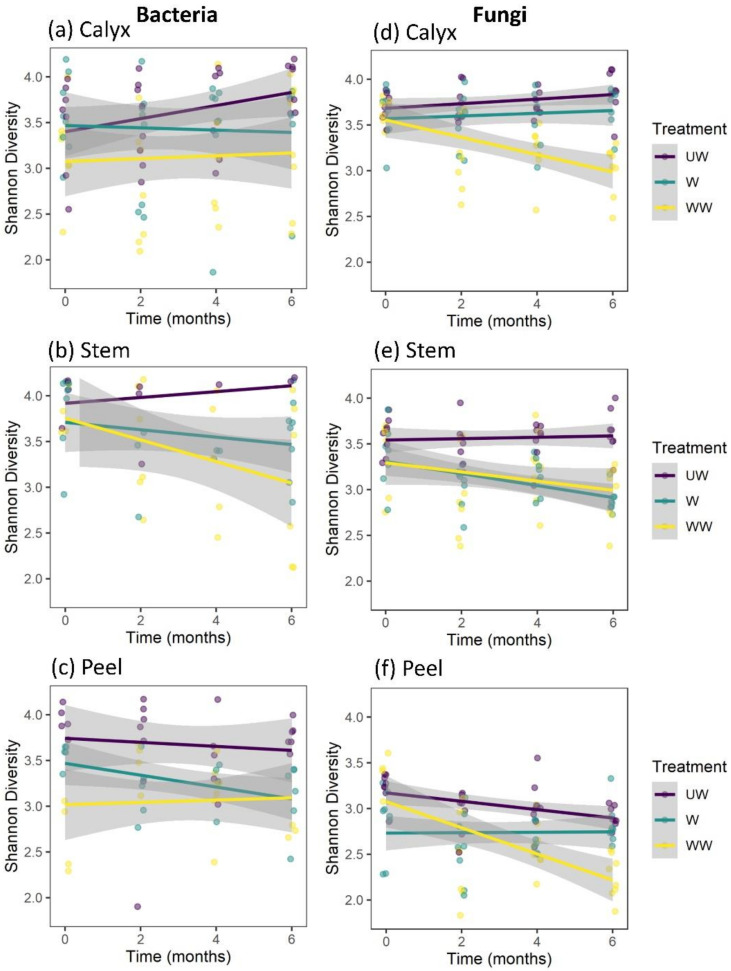
Temporal variations in the bacterial and fungal diversity based on Shannon index at different sampling points (0, 2 m, 4 m, and 6 m) during cold storage of apple calyx-end (**a**,**d**), stem-end (**b**,**e**), and peel (**c**,**f**) tissues.

In our analysis of factors affecting the Shannon diversity of bacteria, a significant effect of treatment and a marginally significant two-way interaction between treatment and tissue were observed. Notably, no interactions between tissue and time, treatment and time, or among treatment, storage time, and tissue were observed (Table 1). Considering the marginal interaction with tissue, and for consistency with other analyses, separate analyses for each tissue type were conducted. Strong effects were observed for postharvest treatment on the diversity of the bacterial community in all tissue types (Table 2), typically with higher diversity on UW fruits (Figure 6). Diversity was also marginally reduced in W and WW stem tissues with increasing storage time (Figure 7b). No interaction was observed between treatment and storage time (Table 2). The differences among treatments were mainly due to a significant reduction in bacterial diversity following washing and washing-waxing, which was particularly evident in peel and stem-end tissues (Figure 6b,c). In contrast, only washing-waxing (WW) significantly reduced diversity in calyx-end tissues, while washing (W) only resulted in a slight reduction in diversity (Figure 6a).

The analysis of factors affecting the Shannon diversity of fungi indicated strong three-way interactions among treatment, storage time, and tissue type, as well as two-way interactions between treatment and tissue type, treatment and time, and a marginal interaction between tissue type and storage time (Table 1). This analysis was followed by separate analyses for each tissue type (Table 2). The results indicated that all tissue types were significantly affected by treatment. Furthermore, storage time significantly reduced the Shannon diversity of stem and peel tissues but not calyx tissues (Table 2, Figure 7d–f). Additionally, significant interactions were observed between treatment and storage time in calyx-end and peel tissues but not stem-end tissue (Table 2). For both the calyx and peel, a significant decrease in fungal diversity was observed with storage time in WW apples (Figure 7d,f). For stem-end tissues, a significant decrease in diversity was observed with storage time in both W and WW apples (Table 2; Figure 7e).

#### 3.3.2. Community Composition

PERMANOVA analysis confirmed and extended the findings on the impact of treatment, time, and tissue-type on the bacterial and fungal microbiome of harvested apples (Table 3). This analysis revealed that the postharvest treatments and storage time had a significant overall effect on bacterial community composition. Furthermore, the interaction between treatment and tissue, tissue and time, as well as treatment and time, had a significant impact on the bacterial community. A significant three-way interaction among treatment, storage time, and tissue was also observed (Table 3).

Considering the observed significant overall effects of time and treatment, pairwise PERMANOVAs were also conducted comparing individual treatments and sampling periods. The results of the pairwise analyses revealed significant differences in the bacterial community composition among UW, W, and WW apples in all tissue types, as well as between most of the sampling points (Table S2). The biggest difference during storage occurred between Time 0 and 6 months, and the least difference between 2 and 4 months.

Fungal community composition was also significantly affected by treatment, time, and the interactions between treatment and tissue, tissue and time, and treatment and time (Table 3). Furthermore, the results revealed a significant three-way interaction among treatment, storage time, and tissue (Table 3). Pairwise comparisons also indicated significant differences in fungal community composition among the three treatments (Table S2). Although the effect of time was significant in almost all of the pairwise comparisons within each tissue type and treatment, the impact was more evident on fungal community composition than it was on bacterial community composition (Table S2). The biggest shift in the fungal community composition during storage occurred between Time 0 and 6 months, and the least difference between 2 and 4 months.

**Figure 8:**
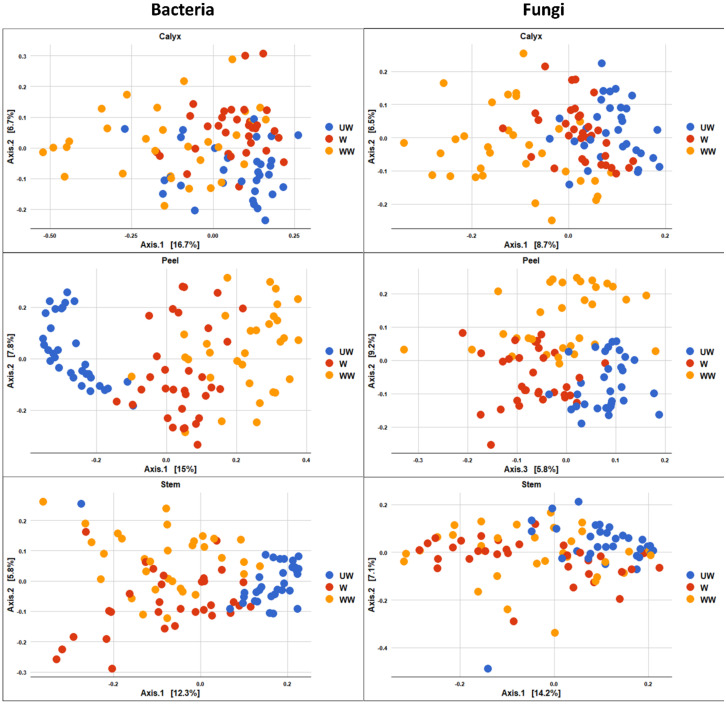
PCoA based on Bray Curtis dissimilarity metrics, showing the distance in the bacterial and fungal communities between postharvest treatment (UW = unwashed, W = Washed, and WW = washed and waxed) in apple calyx-end, peel, and stem-end tissues.

The PCoA plot of bacterial composition attributed to treatment also indicated that UW samples were more segregated, and distinctly clustered from W and WW samples, although the differences were clearer in peel and stem-end tissues (Figure 8). The effect of treatment on the fungal community was similarly most evident between UW and treated fruit (W and WW), with the two groups exhibiting a degree of overlap (Figure 8).

**Figure 9:**
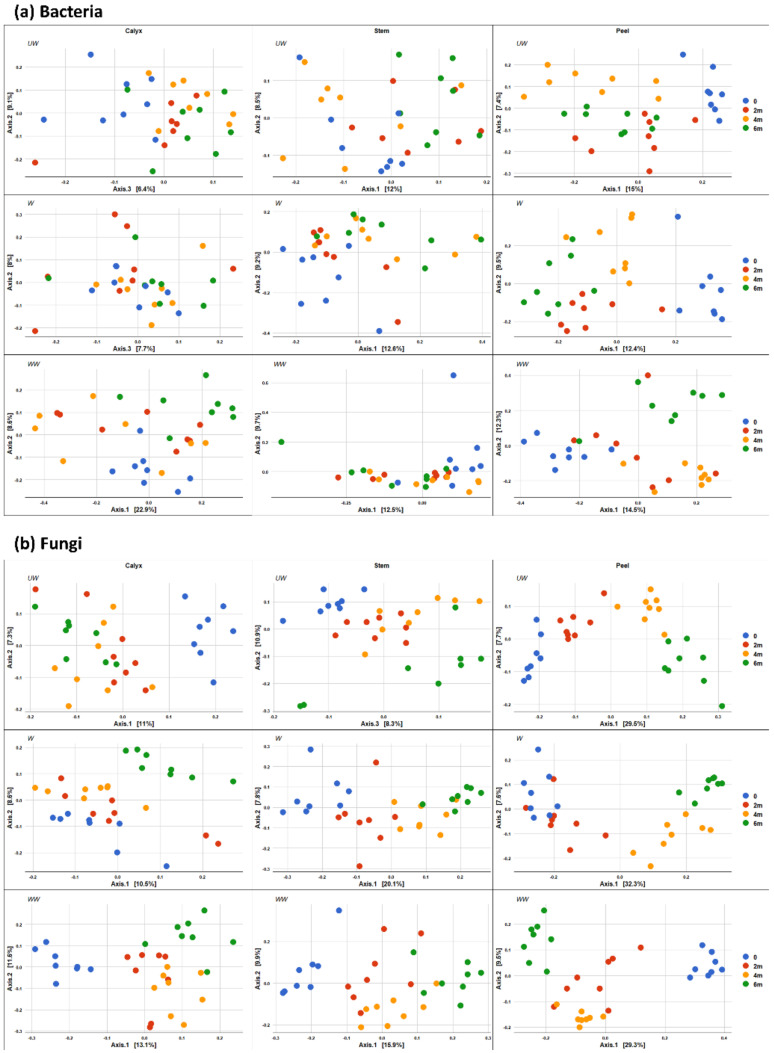
PCoA based on Bray Curtis dissimilarity metrics, showing the distance in the bacterial (**a**) and fungal (**b**) communities between sampling points (0, 2 m, 4 m, and 6 m) in different postharvest treatments (UW = unwashed, W = Washed, and WW = washed and waxed) in apple calyx-end, peel, and stem-end tissues.

The impact of time of storage on the bacterial and fungal community composition is illustrated in Figure 9, in which differences between time points are more evident after 4–6 months of storage. In contrast to the effect of storage time on fungal composition, the effect of storage time on bacterial composition was less evident (Figure 9).

### 3.4. Effect of Postharvest Treatments and Storage Time on the Relative Abundance of Microbial Taxa

**Figure 10:**
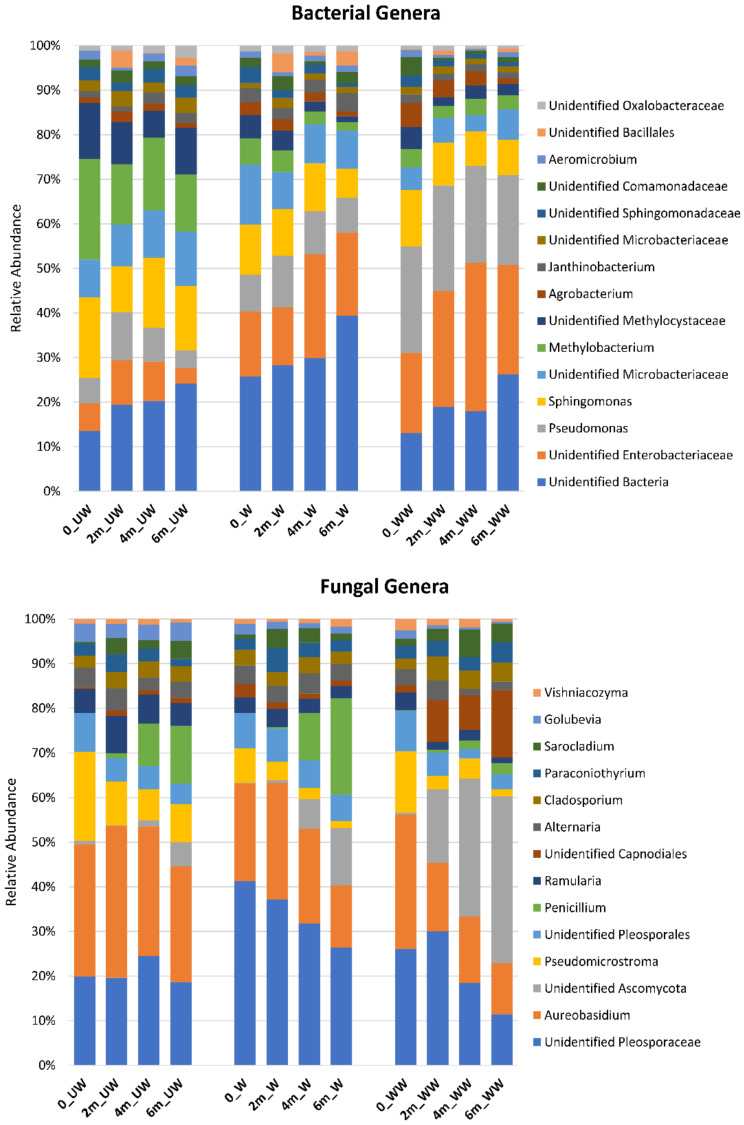
Relative abundances of the most prevalent bacterial and fungal genera present in different sampling points (0, 2 m, 4 m, and 6 m) in different postharvest treatments (UW = unwashed, W = Washed, and WW = washed and waxed).

An overall greater relative abundance of *Rhizobiales* (e.g., *Methylobacterium*) was observed in the bacterial community of UW relative to W and WW fruit, and a higher level of unidentified *Enterobacteriales* and *Pseudomonas* was observed in WW fruit, relative to UW and W fruit (Figure 10). Comparisons of the effect of time and treatment within tissue-types are illustrated in Figure S2. Regarding the fungal community, a higher relative abundance of unidentified *Pleosporaceae* was observed in W fruit, relative to UW and WW fruit, while a higher abundance of unidentified *Capnodiales* was observed in WW fruit, relative to UW and W fruit. In addition, *Aureobasidium* showed a higher relative abundance in UW fruit compared to other treatments. A higher frequency of unidentified *Ascomycota* was also observed in WW, relative to UW and W fruit (Figure 10). The impact of treatment on individual tissues is presented in Table S3. Trends in the individual tissues were similar to those observed for all tissues combined.

A comparative analysis, using the Metacoder version 0.3.4 software [37], revealed that both treatment and time affected the relative abundances of certain bacterial taxa. Bacterial orders that were more abundant in UW fruit than W fruit were *Actinobacteria* (*Thermoleophilia* and *Solirubrobacterales*), *Deltaproteobacteria* (*Bdellovibrionales* and *Myxococcales*), *Bacteriodetes* (*Bacteroidales*), and *Alphaproteobacteria* (*Rhizobiales*, *Sphingomonadales*, and *Rhodobacterales*); see Figure S2a. In contrast, *Gammaproteobacteria* (*Pseudomonadales*) were more abundant in WW fruit than in UW and W fruit. Differences between UW and WW fruit were similar to those between UW and W fruit; however, more diversity was lost, and so a higher relative abundance of *Acidobaceriia* (*Acidobacteriales*), *Planctomycetes*, *Actinobacteria* (*Actinomycetales*), *Sphingobacteria* (*Sphingobacteriales*), and *Alphaproteobacteria* (*Caulobacterales*) was observed. In contrast, an even greater difference in relative abundance was observed in *Gammaproteobacteria* (*Pseudomonadales*) between UW and WW, and W and WW fruit. Lastly, *Betaproteobacteria* (*Burkholderiales*) and *Actinobacteria* (*Actinomycetales*) were more abundant in W fruit than in WW fruit (Figure S2a). Regarding the effect of time (inclusive of all tissue-types and treatments), it appears that there was a gradual enrichment in the relative abundance of *Firmicutes* (*Bacilli*, *Bacillales*); see Figure S2b.

Parallel analyses for fungi also revealed a number of specific fungal taxa affected by treatment and time. Among Basidiomycota, taxa within the *Polyporales*, *Microbotryomycetes* (*Sporidiobolales*), *Tremellales*, *Cystobasiomycetes* and (*Cyphobasidiales*, *Erythrobasidiales*, *Cystobasidiales*) were more abundant in UW fruit, relative to W and WW fruit. Among Ascomycota, taxa within the *Arthoniomycetes* (*Lichenostigmatales*), *Saccharomycetes* (*Saccharomycetales*), and *Chaetothyriales* were more abundant in UW fruit than in W and WW fruit. In contrast, Ascomycota taxa within the Pleosporales were more abundant in W fruit than UW and WW fruit, while taxa within the *Sordariomyecetes* (*Microascales*) were more dominant in WW fruit than UW fruit. Lastly, taxa within the Saccharomycetales were more abundant in WW than in W fruit (Figure S2c). As with bacterial taxa, increasing storage time (inclusive of all tissue-types and treatments) resulted in a gradual decrease in the relative abundance of various fungal taxa (Figure S2d).

The impact of the various treatments and times on individual pathogenic and beneficial taxa are presented in Figure S3. In these figures, the relative abundances of taxa are presented separately within each tissue-type and treatment over six months of storage. The data indicate that treatment did have a differential effect on the relative abundance of the selected taxa. However, several of those taxa responded differently to treatment and/or time, and between tissue types, to those effects. For instance, *Penicillium* was found to have a steady increase in relative abundance over the storage period in all tissue types (Figure S3). In contrast, *Aureobasidium* appeared to be significantly reduced due to waxing treatment and decreased in relative abundance throughout the storage period (Figure S3).

## 4. Discussion

Fruit crops are subjected to various pre- and post- harvest management practices. Postharvest treatments are generally applied to apple fruit to reduce microbial load, minimize spoilage, slow respiration, delay senescence, and improve appearance. During long-term storage, apples are usually kept under conditions whereby the oxygen and carbon dioxide levels in the atmosphere are controlled. While these treatments vary according to the management approach and type of fruit, washing (including the use of a sanitizer) followed by waxing could be considered the minimum treatments applied to the majority of fruit crops. The present study was designed to elucidate the impact of postharvest treatment on the fungal and bacterial communities associated with apple fruit. Since the effect of treatments complexifies with storage time, we also evaluated the impact of low-temperature storage over a period of six months, with sampling occurring at two-month intervals. Given that it was previously demonstrated that different apple tissues harbor distinct microbial communities [10,14], stem-end, calyx-end, and peel tissues were individually sampled and analyzed.

### 4.1. Tissue

The results of the present work reveal that tissue-type is the main factor defining fungal and bacterial diversity and community composition on apple fruit. These results confirm previous studies on the fungi [10] and bacteria [14] present in different apple tissues. The differences reported in the present study were significant in regard to Shannon diversity and community composition, and were evidenced in both fungal and bacterial communities, regardless of the type of treatment or time of storage. Contrary to a previous report (Abdelfattah et al., 2016) where apple peel tissue was reported to exhibit the highest fungal diversity, the present results indicate that calyx-end tissues had higher levels of fungal diversity than stem-end and peel tissues [10]. The differences between the present and previous study may be due to several factors including the use of different cultivars, fruit physiological condition, postharvest treatment, and storage time, as well as the location where the apples were grown. A study by Abdelfattah et al. (2016) was conducted on ‘Red Delicious’, grown in Washington state, but sampled from a local supermarket in West Virginia [10] This fruit had also been stored for at least six months under unknown storage conditions, and subsequently, extensively handled during the process of repacking, shipping, delivery, and being placed on the shelves in the supermarket. In contrast, the current study utilized ‘Enterprise’ apples that were harvested from trees located in an orchard on the grounds of the USDA-ARS in WV, and processed all in a single day. Nevertheless, the microbial community found to be associated with different tissue types in the present study was very similar to what has been described in previous studies [10,14,39]. In the present study, however, we found fewer fungal and bacterial taxa with potential pathogenicity to humans. For instance, *Malassezia*, *Candida*, and *Trichosporon* were previously reported to be present in high proportions in different tissues of apple in fruit obtained from a supermarket [10]. Although the source of these taxa could not be determined in that study, their absence in the present study suggests that they may have been introduced during the intensive handling that occurs during the packing and display of apples in a supermarket.

### 4.2. Treatment

Postharvest treatment was demonstrated to have a strong effect on microbial diversity in all of the different fruit tissues (peel, calyx-end, and stem-end). Notably, treatment was found to reduce fungal and bacterial diversity in similar patterns in the different tissue types. Fruit washing alone resulted in a significant reduction in the microbial diversity present on peel and stem-end tissues, but not calyx-end tissues, with the latter being affected only by the waxing treatment. Washing in general is associated with a significant reduction in microbial loads from plant surfaces [40]. While the main objective of washing and disinfecting fruits and vegetables is to reduce the level of pathogenic inoculum responsible for postharvest decay and/or foodborne human pathogens, such practices may reduce beneficial and pathogenetic microbial load indiscriminately, resulting in lower microbial diversity and/or a significant shift in their community composition. In this regard, the fruit surface (peel) would be most affected, especially considering its smooth waxy surface compared to the rough topography of stem-end and calyx-end tissues that make access of disinfectant solutions more difficult. In particular, calyx-end tissues are cavities filled with remnants of floral tissues (sepals), and as such, represent a protected area that also provides ingress to internal apple tissues. The composition of the fungal and bacterial community composition in different tissues was also affected by the postharvest treatments, with calyx-end tissues being the least affected (probably for the reasons mentioned above). Although postharvest treatment significantly affected both fungal and bacterial community composition, the effect was more evident in the comparison between UW and WW fruits than it was in the comparison between W and WW fruit, indicating that washing alone was enough to result in a significant change in microbial community composition.

### 4.3. Time

The effect of storage time on the microbial community was complex and had a differential impact on fungi vs. bacteria, as well as diversity vs. composition. For instance, storage time had a significant effect on fungal diversity (Shannon diversity) and fungal community composition in all tissue types. The effect of storage time on bacterial diversity, however, was not significant, while its impact on bacterial community composition was only significant at specific time points. These results indicate that fungal diversity was prone to change over time, while the bacterial community was more resilient to shifts in its diversity. The significant shifts in bacterial community composition suggest that it may have undergone species replacement (turnover) and/or a significant change in the relative abundance of the present taxa. These findings agree with previous studies showing a compositional shift in microorganisms, especially fungi, in stored apples [39,41]. The significant shift that occurred in both fungal and bacterial communities, regardless of treatment or tissue type, between T0 and T2, may have been a direct result of storing the apples at low temperature.

In regards to fungi, *Penicillium*, the primary cause of apple postharvest rot, exhibited a significant increase in relative abundance over storage time in all tissue. This may have been due to the aging of apples and the release of exudates (metabolites) on the fruit surface that support the proliferation of this pathogen. In contrast, other natural antagonists such as *Aureobasidium* showed the opposite trend. This is in agreement with a previous study of stored apples [39]. While the specific mechanisms behind these changes are unclear, the compositional changes have pathological significance in relation to the development of decay and natural antagonisms during prolonged storage periods.

Macarisin et al. (2019) reported that washing and waxing apple fruits has a beneficial effect on the survival of the foodborne pathogen *Listeria monocytogenes*, and suggested that this may be partially attributed to the wax providing a protective environment [20]. Data from the present study indicate that a reduction in the overall diversity of the microbial community and suppressive effect on the relative abundance of individual taxa may also have contributed to a favorable environment for the growth of *Listeria* by altering competitive or trophic interactions that the foodborne pathogen has with other microbial taxa in the community. Our data also indicate that the absence of any washing or washing/waxing treatment (i.e., letting the ‘natural microflora’ flourish) did not have a suppressive effect on the relative abundance of common decay pathogens. More specifically, allowing the ‘natural microflora’ to flourish did not have a suppressive effect on common decay pathogens. Notably, however, the mere presence of a population of a pathogenic microbe does not necessarily indicate the potential for infection. Other members of the fruit microbiota may still have a suppressive impact on infection while not impacting the level of pathogen inoculum. In this regard, a recent study of *Erwinia amylvora*, the causal agent of fire blight, on inoculated flowers indicated that despite the pathogen being present in very high numbers and favorable environmental conditions, less than half of the inoculated flowers became infected [42]. Additionally, the fruit microbiome in the current study was conditioned by the outcome of standard conventional orchard practices involving the use of synthetic chemicals (fungicides and insecticides), and should be compared with the ‘natural microflora’ that results from no interventions, or at least from organic management practices, to determine the potential impact of those microorganisms on disease development. A deeper understanding of the fruit microbiome will be needed to develop strategies that can be used to favor the establishment of a microflora that is disease suppressive, be it through genotype selection, management practices, the administration of a biocontrol agent, the use of a synthetic community, or the application of compounds that have a beneficial impact on the fruit microbiome.

## 5. Conclusions

In the present study, the effect of postharvest treatments (washing and waxing) and low-temperature storage on the fungal and bacterial communities associated with different apple tissues (calyx-end, stem-end, and peel) were examined. The results indicated that tissue-type is the main factor defining fungal and bacterial diversity and community composition of apple fruit. Within those tissue types, however, both postharvest treatments and low temperature storage had a significant impact on fungal and bacterial diversity, as well as community composition. The greatest temporal shift in diversity occurred during the first two months of low-temperature storage; however, this was more evident for fungi than for bacteria.

The biological control of postharvest diseases has been comprehensively explored over the past 30 years. Success in the commercialization of products that are consistently effective and reliable, and are widely used, however, has not been forthcoming. Droby and Wisniewski (2018) suggested that the relationship between biocontrol agents, the resident microbiome, and the effect of management practices is a missing component in the current understanding of biocontrol systems [17]. While several studies have focused on the effect of management practices on the fruit microbiome, the majority have overlooked interactions between key aspects of fruit postharvest handling, and lacked a rigorous statistical analysis of the main effects and interactions between the various postharvest treatments on the fruit microbiome. In this regard, the present study provides a comprehensive analysis of postharvest treatments (washing and waxing) and low-temperature storage (2 °C for six months) while accounting for their interactions, as well as the spatial variation within an individual fruit on the diversity and composition of fungi and bacteria in harvested apples. As such, it can serve as a model for future microbiome-related studies of harvested fruit.

## Figures and Tables

**Table 1:** Model results using anova test on the effects of treatment, tissue, and storage time, and their interactions with the Shannon diversity of bacteria and fungi on apple fruits.

	Bacteria		Fungi	
	F Value	Pr (>F)	F Value	Pr (>F)
Treatment	26.973	2.56 × 10^−^^11^	56.58	<2 × 10^−^^16^
Tissue	35.98	1.96 × 10^−^^14^	165.926	<2 × 10^−^^16^
Time	1.831	0.1773	20.982	7.16 × 10^−^^6^
Treatment × Tissue	2.068	0.0855	3.995	0.00365
Treatment × Time	1.407	0.2469	12.049	9.85 × 10^−^^6^
Tissue × Time	1.455	0.2353	2.417	0.09116
Treatment × Tissue × Time	0.726	0.5751	3.609	0.00696

**Table 2:** Model results using anova test on the effects of treatment and time on bacterial and fungal Shannon diversity on apple’s calyx-end, stem-end, and peel.

		Calyx		Stem		Peel	
		F Value	Pr (>F)	F Value	Pr (>F)	F Value	Pr (>F)
Bacteria	Treatment	7.564	0.000925	10.577	7.49 × 10^−^^5^	16.051	2.02 × 10^−^^6^
	Time	0.231	0.631866	3.574	0.0619	1.036	0.312
	Treatment × Time	0.608	0.546641	2.189	0.1179	0.325	0.724
Fungi	Treatment	30.518	8.07 × 10^−^^11^	22.535	1.25 × 10^−^^8^	17.097	5.60 × 10^−^^7^
	Time	1.977	0.16317	6.58	0.012	14.15	0.000307
	Treatment × Time	10.119	1.10 × 10^−^^4^	2.575	0.0819	7.898	0.00071

**Table 3:** PERMANOVA results on testing the effects of tissue, treatment, and time, and their interactions on bacterial and fungal communities of apple fruits. The comparisons were based on Bray Curtis dissimilarity, and *p*-values were calculated using the adonis function in vegan and corrected using the FDR method.

	Bacteria		Fungi	
	R^2^	Pr (>F)	R^2^	Pr (>F)
Tissue	0.19293	0.001	0.16309	0.001
Treatment	0.06442	0.001	0.04962	0.001
Time	0.0382	0.001	0.09419	0.001
Tissue × Treatment	0.04249	0.001	0.02634	0.001
Treatment × Time	0.02803	0.001	0.03742	0.001
Tissue × Time	0.0275	0.001	0.03841	0.001
Tissue × Treatment × Time	0.03766	0.002	0.04455	0.001

## Supplementary Materials

The following are available online at https://www.mdpi.com/2076-2607/8/6/944/s1, Table S1: Sequencing summary of the 16S and ITS amplicons showing the total number of samples, quality-filtered sequences, and the number of ASVs assigned to those sequences, Table S2: Statistical comparison of community composition between apple tissues, treatment, and storage time according to pairwise PERMANOVA. *p* values were corrected using FDR method, Table S3: Differential abundant bacterial and fungal taxa between apple tissues: Calyx-end, Peel, and Stem-end (Sheet 1) and among treatments: Unwashed, Washing, Washing-Waxing on apple tissues (sheet 2) according to Kruskal-Wallis test, Figure S1: PCoA plots based on Bray Curtis dissimilarity metrics, showing the distance in the bacterial and fungal communities between apple calyx-end, peel, and stem-end tissues in different treatments (UW = unwashed, W = Washed, and WW = washed and waxed) at each sampling points (0, 2 m, 4 m, and 6 m), Figure S2: Heat trees showing differential abundance of bacterial (a and b) fungal (d and c) due to postharvest treatments and storage time. Tree structures represent the taxonomic hierarchy of Amplicon sequence variants (ASVs) classifications up to order level. Tree branches are correlated with order abundances, Figure S3: Response of some pathogenic and beneficial genera to postharvest treatments (UW = unwashed, W = Washed, and WW = washed and waxed) at sampling points (0, 2 m, 4 m, and 6 m).
